# How to design equitable digital health tools: A narrative review of design tactics, case studies, and opportunities

**DOI:** 10.1371/journal.pdig.0000591

**Published:** 2024-08-22

**Authors:** Amy Bucher, Beenish M. Chaudhry, Jean W. Davis, Katharine Lawrence, Emily Panza, Manal Baqer, Rebecca T. Feinstein, Sherecce A. Fields, Jennifer Huberty, Deanna M. Kaplan, Isabelle S. Kusters, Frank T. Materia, Susanna Y. Park, Maura Kepper

**Affiliations:** 1 Behavioral Reinforcement Learning Lab (BReLL), Lirio, Inc., Knoxville, Tennessee, United States of America; 2 School of Computing and Informatics, University of Louisiana at Lafayette, Lafayette, Louisiana, United States of America; 3 College of Nursing, University of Central Florida, Orlando, Florida, United States of America; 4 Department of Population Health, NYU Grossman School of Medicine, New York, New York, United States of America; 5 Department of Psychiatry and Human Behavior, Warren Alpert Medical School of Brown University, Providence, Rhode Island, United States of America; 6 Weight Control and Diabetes Research Center, The Miriam Hospital, Providence, Rhode Island, United States of America; 7 Neamah Health Consulting, Boston, Massachusetts, United States of America; 8 AIHealth4All Center for Health Equity using Machine Learning and Artificial Intelligence, University of Illinois at Chicago, Chicago, Illinois, United States of America; 9 Department of Psychological and Brain Sciences, Texas A&M University, College Station, Texas, United States of America; 10 Fit Minded Inc., Phoenix, Arizona, United States of America; 11 Department of Family and Preventive Medicine, Emory University School of Medicine, Atlanta, Georgia, United States of America; 12 Department of Spiritual Health, Woodruff Health Science Center, Emory University, Atlanta, Georgia, United States of America; 13 Department of Clinical, Health, and Applied Sciences, University of Houston-Clear Lake, Houston, Texas, United States of America; 14 Center for Medical Ethics and Health Policy, Baylor College of Medicine, Houston, Texas, United States of America; 15 Otolaryngology and Population Health, University of Kansas Medical Center, Kansas City, Kansas, United States of America; 16 Radiant Foundation, Salt Lake City, Utah, United States of America; 17 Prevention Research Center, Brown School, Washington University in St. Louis, St. Louis, Missouri, United States of America; National Tsing-Hua University: National Tsing Hua University, TAIWAN

## Abstract

With a renewed focus on health equity in the United States driven by national crises and legislation to improve digital healthcare innovation, there is a need for the designers of digital health tools to take deliberate steps to design for equity in their work. A concrete toolkit of methods to design for health equity is needed to support digital health practitioners in this aim. This narrative review summarizes several health equity frameworks to help digital health practitioners conceptualize the equity dimensions of importance for their work, and then provides design approaches that accommodate an equity focus. Specifically, the Double Diamond Model, the IDEAS framework and toolkit, and community collaboration techniques such as participatory design are explored as mechanisms for practitioners to solicit input from members of underserved groups and better design digital health tools that serve their needs. Each of these design methods requires a deliberate effort by practitioners to infuse health equity into the approach. A series of case studies that use different methods to build in equity considerations are offered to provide examples of how this can be accomplished and demonstrate the range of applications available depending on resources, budget, product maturity, and other factors. We conclude with a call for shared rigor around designing digital health tools that deliver equitable outcomes for members of underserved populations.

## Author summary

Designers and developers of digital health tools, such as apps or telehealth services, have an opportunity to improve how their products support health equity. After the COVID-19 pandemic, the murder of George Floyd, and the passage of the 21st Century Cures Act, there are several forces in American society bringing attention to historical healthcare inequities and momentum to address them as we develop new products. We recommend practitioners start by consulting health equity frameworks to identify which dimensions, such as race, age, or health status, are most relevant to their product. From there, practitioners can choose established design tools like the Double Diamond Method, the IDEAS framework and toolkit, or community collaboration techniques like participatory design for their product development process and deliberately infuse a health equity focus. To demonstrate how that is possible across contexts, we offer case studies that range from lower-intensity equity research to enduring partnerships with community organizations serving underrepresented user groups. The goals of this paper are to equip practitioners to achieve more equitable outcomes via digital health tools and to contribute to standard methods of adopting an equity focus in digital health work.

## Introduction

The current backdrop in the United States includes a mix of supportive legislation and major historical events that amplify the ongoing effects of historical inequity. In 2020, the 21st Century Cures Act (“Cures Act”) was released by the Office of the National Coordinator for Health Information Technology (ONC). These rules call for improvements to how health data is collected and shared in the hopes of accelerating digital innovation [1]. The Cures Act creates conditions for more widespread adoption of digital health by historically underserved groups and establishes a mandate for those developing digital health tools to take specific steps toward more equitable design. Meanwhile, national crises such as the COVID-19 pandemic and the murder of George Floyd have sparked renewed energy toward addressing historical inequities in healthcare [2,3]. It is beyond time for those who create digital health tools to harness that energy into action.

We offer several strategies to promote health equity considerations throughout the digital health tool design process, as well as case study examples of how these processes support user experience and outcomes, with the goal of equipping those developing digital tools with methods to design more equitable outputs. These strategies may benefit what we term *practitioners*, or anyone developing a digital health tool, including academics, industry organizations, designers, behavioral scientists, and others. Due to the number of digital health technologies utilizing behavioral science approaches, and the authors of this paper including behavioral scientists in their number, behavioral science is well represented in the methodologies and case studies presented. Our focus is on how the **design** phase of product development can promote health equity, recognizing that it is only one of the target areas for equity consideration in the development and implementation of digital health tools [4].

## Defining “Digital Health Tools”

We define *digital health tools* as any product that uses information and communication technologies such as computing platforms, software, connected devices, or sensors to help people or their providers support their health. Digital health tools, sometimes referred to as mobile health (mHealth), health information technology (HIT), wearable devices, or telehealth and telemedicine, may provide positive benefits related to access to and cost of care and quality of outcomes. Their intended users may include people in a consumer or patient context, providers such as nurses, doctors, or coaches, and nonprofessional caregivers. We focus on digital health tools which are patient- or caregiver-facing, rather than intended for provider or organizational use, with the belief that the greatest opportunity to improve health equity comes from directly supporting the people who experience inequity.

Digital health tools address a breadth of topics, including the diagnosis of diseases, delivery of treatment, support for behavioral changes, or improvement of healthcare delivery and health promotion. Digital health tools span from highly regulated solutions such as “digital therapeutics” to consumer products such as the Apple watch or Noom’s weight management services, to low-code apps developed for research projects or pilots [5,6]. The techniques in this paper can be applied to digital health tools across the full spectrum of regulation, technological sophistication, and application domains.

## Defining “Health Equity in Digital Health”

Health equity can be considered both a process and an outcome. Health equity is achieved with the attainment of the optimal level of health for all members of society through valuing individual and social group differences, continuous efforts to address avoidable inequity and social injustices, and eradication of disparities in health and access to healthcare [7]. Sometimes health equity in digital health is mistakenly perceived as providing a unified or standardized digital health tool to all segments of the society. However, equal services do not necessarily lead to equity, given that underserved groups may have different needs to achieve equal outcomes. For practitioners, this has implications for every part of the digital health product life cycle. Achieving health equity requires digital health tools to be created with a systemic lens that considers all factors related to their users’ ability to seek health services, properly utilize health products, or achieve optimal health outcomes by using these tools. Health equity frameworks can provide that lens that ensures that factors causing inequity within the intended audience are addressed within the design process. These factors include sex and gender, age, ethnicity, race, national origin, language, ability, religion, sexual orientation, educational level, health status, location of residence, broadband internet availability, socioeconomic status (SES), and immigration status [8].

Digital health also offers unique challenges compared to analog approaches in that technology itself has equity implications. For example, digital health tools that use artificial intelligence (AI) may be subject to potential bias related to algorithm design [9]. There also may be decisions around what equipment or technology is necessary for people to use a digital health tool; the use of modalities such as virtual or augmented reality or even smart watches may inadvertently exclude members of disadvantaged groups from the benefits of digital health [10], as indicated by research showing a negative relationship between SES and access to digital health [11]. The “Digital Divide” refers to challenges impacting availability, affordability, accessibility, quality, security and privacy, and digital literacy that ultimately perpetuate health disparities and widen the healthcare inequity gap [12]. While digital modalities such as email and text message [13,14] are widely available across income and educational levels, access to the internet and devices may be limited or inconsistent due to effects of digital redlining [15], such that some digital health tools may exacerbate disparities [16]. And even when access exists, some people may have low levels of digital self-determination reflecting a lack of knowledge about how to effectively engage with technology [17] (i.e., digital literacy). Finally, delivering equitable digital health tools requires consideration not just of health literacy and numeracy, but also digital health literacy, which looks at the ability to evaluate and use health information delivered via technology [18,19] and may not cleanly overlap with other literacy constructs. Research suggests digital health literacy is quite variable, particularly among underserved populations [11,19].

That said, there is a reason why digital health has appealed to equity-focused practitioners. Internet access is increasingly ubiquitous, with 93% of adults in the US using the internet in 2021, up from 90% in 2019 [20]. This increase in use, corresponding to the onset of the COVID-19 pandemic, includes increased uptake of digital health interventions [21]. There is promise for digital health, if equity can be addressed.

## Focus on designing for equity

The design phase determines whether a digital health intervention will be able to address or overcome disparities. Early decisions about a product’s components and functionality matter for how it can ultimately be used, as well as its relevance and acceptability [22]. Design is typically a cross-functional phase with inputs from people not formally trained to consider health equity such as UX and interaction designers, software engineers, or content writers. When trained practitioners working on interventions raise health equity considerations, other team members can be made aware of potential downstream impacts of their design decisions and coached to recognize opportunities for equity. Inappropriate designs can lead to problems that will be difficult to rectify in later stages of development, resulting in products that are neither effective nor widely adopted. Iterative design phases (such as new releases for digital products [23]) offer another opportunity to enhance the delivery of equitable support after a product’s initial release. Finally, for some organizations, an explicit focus on health equity could have implications for funding opportunities and product-market fit.

A recent scoping review of the equity impact of digital health design practices found key gaps around rigorous and consistent application of design methodologies, scaling digital health tools beyond pilots, and embedding equity-focused evaluation mechanisms [24]. While the implementation and operation of digital health interventions must also support equitable outcomes, their ability to do so is rooted in whether the design process considers the context in which it will be used and mechanisms for scale and evaluation. Similarly, while measurement of equitable outcomes is critical for ensuring a tool delivers them, no amount of measurement will make a poorly designed tool work. A focus on *designing* for health equity may therefore have outsized impact on results.

## Frameworks for understanding determinants of health equity

Given the variety of factors that can negatively impact health equity, practitioners must systematically evaluate how to position their work to address the most relevant ones. To support the identification and overcoming of equity challenges, we highlight several frameworks (PROGRESS-Plus, eHLF, DHE, HEIF) that address different phases of the development and implementation of digital health interventions, as well as end-user characteristics related to equity. Practitioner teams can reference these frameworks early in their process and use them to identify specific factors to target in the interest of equity.

### PROGRESS-Plus

The PROGRESS-Plus framework enumerates individual and relational characteristics that might lead to health inequity including **P**lace of residence, **R**ace/ethnicity/culture/language, **O**ccupation, **G**ender/sex, **R**eligion, **E**ducation, **S**ocioeconomic **S**tatus (SES), Social capital, **Plus** additional characteristics such as age, disability, relationships with others, and events that create disadvantages [25,26]. PROGRESS-Plus and similar frameworks can help digital health designers systematically account for the broad set of characteristics of end users that impact health equity.

### eHLF

The eHealth literacy framework (eHLF) offers a conceptual model for characterizing the interaction and relation between individuals and systems via digital health technologies [27]. eHLF describes 7 domains of e-health literacy, defined as the capabilities and resources that people must have to benefit from digital health, and can be used to evaluate e-health literacy for both patients and healthcare professionals. The 7 domains are mapped by whether they characterize individuals, systems, or the interaction between the two, and whether they are internal or external. The domains include: ability to process information, engagement in one’s own health, ability and motivation to engage with digital services, feeling safe and in control, and access to effective digital services that appropriately address individual needs. There is an interaction between individual and system factors across the 7 different eHealth literacy domains such that the factors in the eHLF model intersect with some social determinants of health such as employment and education [28]. With the development of a validated instrument to measure the 7 eHLF dimensions, the framework can be applied to evaluate interventions and how well they are implemented and adopted [29].

### DHE

The Framework for Digital Health Equity (DHE [8]) goes beyond the eHLF with its focus on digital literacy to present a more comprehensive approach to developing equitable digital health tools. DHE builds upon the health disparities framework developed by the National Institute on Minority Health and Health Disparities (NIMHD [30]) by incorporating social, environmental, and structural factors and examining digital determinants of health (DDoHs) at various levels of influence. DDoHs are factors related to digital technology that can influence access to digital healthcare, and, consequently, impact health and well-being. The framework postulates that DDoHs exist at 4 levels (individual, interpersonal, community, and societal), each of which must be considered to mitigate health disparities with digital health.

Considering these levels offers a structured method for identifying and addressing needs and challenges faced by underserved communities. For example, DDoHs at the individual level include factors that affect a person’s capacity to use and adopt digital health solutions. Similarly, personal relationships may influence engagement with digital health technologies; caregiver scenarios, for example, imply a need to design applications that accommodate multiple users with patient consent. Community-level DDoHs include digital technology and infrastructure factors that affect individual health outcomes, such as the availability of high-speed internet. Such resource limitations may necessitate enabling transmission of health information over low-bandwidth connections (e.g., voice- instead of video-based services). Finally, societal factors exist beyond the control of individuals and their communities and are shaped by influences such as government, corporations, algorithms, norms, and ideologies. For example, if risk assessment tools encode societal racial biases, practitioners could redevelop algorithms to ensure they do not include race as a factor or adjust them to correct such biases [31,32].

### Health Equity Implementation Framework (HEIF)

While the previously described frameworks may guide the design and dissemination of digital health solutions, implementation science frameworks offer valuable approaches for addressing health equity through a focus on the context and manner in which interventions will be used [33,34], which should be considered in the design phase. The Health Equity Implementation Framework (HEIF [35,36]) integrates 2 established frameworks: the Healthcare Disparities Framework [37], which addresses factors underlying inequities in care, and the i-PARIHS [38], a widely used implementation determinants framework. The HEIF maps implementation determinants (e.g., features of the intervention and its recipients) according to domains known to affect equity in healthcare. An implementation analysis using the HEIF elucidates how practitioners can simultaneously address disparities factors (e.g., digital literacy, SES, access to technology) and interlocking implementation factors (e.g., beliefs about technology, accessibility and acceptability of interventions). Given that issues of health equity are inextricable from issues of implementation when it comes to digital health technologies, frameworks like HEIF may be necessary for comprehensive examinations of factors impacting accessibility and adoption in digital health.

## Methods and techniques for equitable design

Equitable design, rooted in equity-centered thinking, demands a conscious and intentional approach prioritizing inclusivity, collaboration, and attentiveness to the diverse needs of marginalized communities [24]. It requires a multifaceted strategy that addresses the intricacies of human–technology interactions, societal influences, and the needs of diverse influencers in context and goes beyond the scope of traditional human-centered design by addressing the social and ethical dimensions of technology in concert with co-design principles [39]. A broader examination of power dynamics is needed to ensure members of underserved groups can access and meaningfully use digital health tools.

While equitable design has a noble aspiration, several challenges make it difficult in practice [24]. A significant issue is gaining access to and recruiting members of the underserved groups in the formative design stages [16]. Access may be difficult due to factors such as geographical isolation, a lack of resources to participate in design activities, or community wariness of outsiders stemming from historical exploitation or neglect. Even if access is gained, recruiting members from these groups to actively participate in design activities can be challenging due to cultural, linguistic, or socioeconomic factors. For example, individuals may not have the time to participate due to work commitments or not see value in contributing. Another complication is that competing pressures from funders or organizational leaders may render it infeasible for practitioners to invest the substantial time and resources needed to develop a nuanced understanding of contextual factors that influence the target audience. Thus practitioners may not always fully understand the needs, capabilities, and preferences of underserved communities to create suitable digital health tools. These barriers, combined with industry pressure, cognitive biases, and a lack of awareness, may tempt practitioners to follow existing design paradigms that are not inclusive or equitable. Finally, there is the challenge that many existing design techniques do not explicitly call for a consideration of equity as part of their use, which makes it easy to follow a “correct” process without delivering equitable outcomes.

That said, practitioners can use many techniques from human-centered design that offer concrete methods to address health equity and can be integrated with the product design process. These design techniques are not equity focused unless practitioners deliberately insert a focus to understand and promote equity, so explicit intention on the part of design teams is a prerequisite. Several similar frameworks, such as IDEO’s “inspiration, ideation, implementation” [40] and Stanford d.school’s “empathize, define, ideate, prototype, test [41]” are prevalent in the design industry thanks to the evangelism of their originators. Despite differences in labels and stages, these frameworks share the core principle of centering the design process around individuals with firsthand experience to ensure that the final product or resource is well suited for its intended users. This focus on the end user creates the opportunity for practitioners to work with members of underserved groups in the service of equity. Below, we discuss approaches from human-centered design and how they can accommodate concrete methods for practitioners to create more equitable digital health tools.

### The Double Diamond

The British Design Council introduced the Double Diamond model to harmonize design approaches and management practices [42], and later updated it to increase emphasis on the need for both divergent and convergent thinking at every stage of the design process and insert principles for collaboration, inclusivity, and leadership [43]. The model introduced 4 stages alternating between divergent and convergent thinking: discover, define, develop, and deliver. Divergent stages include activities such as information gathering, hypothesis generation, and brainstorming. Conversely, convergent stages include prioritizing findings, establishing specific requirements, and monitoring and evaluation. An analysis of topics discussed by design teams throughout the project lifespan suggests the Double Diamond model accurately depicts real-world design approaches [44].

The Double Diamond model introduces a level of structured deliberation for practitioners and opportunities to insert insights from members of underserved groups, thereby incorporating the needs of diverse audiences [45]. The inclusion of research from underserved groups, even if they will ultimately be a small part of the target users for the digital health tool, can help ensure the right “ingredients” are included to serve a broad audience. The divergent thinking stages emphasize deviations from the status quo, which may help serve health equity when typical ways of approaching a problem were designed for advantaged groups. The convergent thinking stages, on the other hand, focus on refining choices through targeted feedback from intended users, streamlining the design to meet their specific needs effectively. By clearly delineating goals of user engagement in the design process, practitioners can focus their research efforts to enhance their productivity and respect the time and resources of the target audience. Indeed, literature confirms that the use of this model can shorten the design time cycle [46]. The Double Diamond also accommodates the use of secondary research, particularly in the discover and design phases, which can be advantageous for teams without direct access to potential end users or other practical constraints on their primary research capabilities.

The model’s emphasis on iterative feedback loops is key to the ongoing refinement of solutions, drawing on fresh insights and feedback from diverse voices within marginalized communities. This facilitates ongoing relevance and fairness of the solutions, as it allows practitioners to continually adapt and respond to evolving needs and contexts of these groups. For example, understanding people’s experiences before and after engaging with a digital health tool could help account for the context in which underserved people engage with digital health and offer opportunities to better support their needs through design [47].

### IDEAS framework and toolkit

The Integrate, Design, Assess, and Share (IDEAS) framework and toolkit incorporate user-centered design elements for intervention design [48]. While IDEAS maps neatly onto the Double Diamond, it also explicitly incorporates behavior change theories and includes a toolkit with concrete processes to support the development of equitable digital health interventions [49–51]. IDEAS is composed of 10 phases (empathize, specify, ground, ideate, prototype, gather, build, pilot, evaluate, and share) that are grouped into 4 overarching stages (see Table 1). The IDEAS framework has been used across a number of health behavior design applications ranging from apps to increase physical activity in college students [52] and adult Latinas [53] to interventions to aid cancer survivors [54].

**Table 1:** An overview of the stages and phases, along with component activities, of the IDEAS framework. Adapted from Mummah and colleagues [48].

Stage	Phase	Activities
**Integrate** insights from users and theory	Empathize with target users	Craft research questions; focus inquiry on behaviors, technology usage and attitudes, needs, motivations, and behavioral determinants
	Specify target behavior	Translate behavioral goals into specific target behaviors
	Ground in behavioral theory	Reference behavioral theories and identify strategies to include in the tool
**DEsign** and iterate with user feedback	Ideate implementation strategies	Brainstorm ideas to support user experience and winnow down
	Prototype potential products	Develop prototypes, share within the team to improve, and winnow down to the most promising
	Gather user feedback	Conduct user research to gain insights for product improvement
	Build minimum viable product (MVP)	Build initial intervention and include app analytics to guide iterations
**Assess** rigorously	Pilot potential efficacy and usability	Conduct a small scale evaluation with users and analyze usage behavior
	Evaluate efficacy in RCT	Complete more rigorous outcomes research that includes investigation of mechanisms of action
**Share**	Share intervention and findings	Publish findings, partner with other organizations for dissemination, and continue iterating on tool

With the first 2 stages of the IDEAS framework, the aim is to gather information from users and important influencers about unmet needs and target behaviors of interest and later to elicit feedback on the use of the intervention technology. These are particularly important stages for applying the equity considerations identified using frameworks like PROGRESS-Plus, eHLF, DHE, and HEIF to make sure that diverse voices are included. While the IDEAS framework has been used to design for medically marginalized populations [48,55], it does not directly reference equitable design. Therefore, it is incumbent on the practitioners using the framework to ensure attention to equity is incorporated throughout all design phases.

### Community collaboration techniques

Collaboration with community-embedded organizations adds layers of imperative complexity to the design process. This approach empowers community members as collaborators, adding perspectives that ground research in real-world context and lived experiences. The input of multiple individuals and influencer groups leads to product designs acceptable to horizontal (actual communities) and vertical (community leaders) user groups. Benefits include the improved likelihood that the final product will be considered acceptable, need minimal adaptation for use, and promote sustainable use and equitable health outcomes [56].

Collaboration with community-embedded organizations and providers can significantly mitigate the challenges of equitable design, particularly in gaining access to and recruiting members of underserved groups [57]. These entities often have established trust and rapport within the community which can help overcome wariness of outsiders. They can also provide valuable insights into the specific cultural, linguistic, and socioeconomic factors that might affect participation, thereby helping to tailor recruitment strategies more effectively. For example, in the development of a digital health intervention for low-income Mexican-American women who work on farms, leveraging the help of a community activist facilitated access and built trust within the community [58]. In contrast, lack of initial trust building with a community-based prenatal care organization led to low uptake and ultimate abandonment of the project in another study [59].

Collaborating with community-embedded organizations and providers can serve as a bridge to target communities. This facilitates a more inclusive and effective design process and direct access to the populations with the most need for health promotion and treatment. One final best practice that practitioners can enact when working with community-based partners is to always share study results back to the community organization and providers and reinforce the value they provided to the project. This practice helps to facilitate viable long-term research relationships with community partners and build trust that data is being used to serve the community as well as research.

Multiple design techniques focus on collaboration with community organizations and influencers to design solutions for specific community needs [60]. Table 2 offers an overview of some of them.

**Table 2:** An overview of several community collaboration design techniques that can be used to improve health equity.

Technique	Description
Participatory Design [60]	A designer-led process using participatory processes such as co-design workshops and interviews to elicit experience insights and product requirements from end users.
Action Design Research [61,62]	Enlists community members as direct design collaborators on the strength of their lived experience; characterized by iterative planning and action cycles with participating community members.
Asset-Based Community Development [63]	Empowers community members to drive solutions by identifying and making use of existing but unrecognized or under-used assets such as individuals, institutions, place-based assets, or relationships.
Service Learning [64,65]	Pairs designers with community organizations for mutual benefit; designers work alongside and learn from community experts and context, and have structured opportunities to channel learnings into design outputs.
Stakeholder-Driven Design [66]	Incorporates insights from community members, leaders, and other influencers to understand the problem space and design adequate solutions.
Collaborative Prototyping [67,68]	Solicits feedback from community members on product prototypes or existing products to refine and iterate the designs.

*Participatory Design/Co-design*: In participatory design, members of the communities for whom a product or service is intended are brought directly into the design process as co-creators. Non-designers are enlisted alongside experts for input and hands-on product work, in recognition that end users embody a design competence that can be tapped to develop or improve that product [69]. One of the distinguishing characteristics of participatory design is the removal of typical power relationships where the practitioner has an advantage over the participant [70]. Recent frameworks examining the protection of vulnerable individuals engaging in participatory design emphasize that lived experience is in fact expertise and should be regarded as such [71]. Participatory design has roots in Lewin’s “action research,” which was undertaken in partnership with people experiencing the phenomena being studied [72]. At its most extreme, participatory design enlists future users as full partners in the design process; in reality, participatory design typically offers inputs to the design process that are then evaluated and interpreted by expert practitioners [73]. Design teams may also choose to use participatory design methods to inform discrete aspects of their process [74] or gather user requirements [75].

Participatory design facilitates the expression of *latent needs* [76] that people may not be aware of through introspection or able to articulate, but which nonetheless may heavily influence preferences and use of digital health tools. It builds on traditional research methods of self-report and behavioral observation to incorporate activities in which people create research artifacts. The toolkit of participatory design includes creating tangible items, storytelling about them, and acting or playing [77]. The toolkit can be applied across design activities such as field studies, workshops, collaborative prototyping, or evaluation [78]. Ideally, participatory design activities are facilitated by experts with an eye to participants’ level of ability and ensuring a safe space for active engagement [79].

Participatory design can help mitigate issues of recruitment of underserved groups by creating a collaborative environment where users feel safe and empowered to share their thoughts and ideas. It also ensures that the final product is not only technically feasible (as determined by practitioners) but also culturally and contextually appropriate, thereby increasing the likelihood of adoption and sustained use. In fact, participatory design methods do seem to yield more appealing and well-rated products for their end users [80].

The Double Diamond model, IDEAS framework and toolkit, and methods of community collaboration are useful for practitioners to reference in the conceptualization and development of digital tools to ensure equity issues are carefully considered. In the next section, we offer several case studies where practitioners have used these and similar methods with equity considerations to design digital health tools.

## Digital health equity case studies

In this section, case studies of digital health tools that have been developed using the design methods previously described illustrate the practical application of these methods in real-world scenarios. Each case study showcases a unique approach to design, reflecting the diverse contexts and populations these methods can serve. These examples provide insights into the versatility and impact of these design approaches in fostering innovation, inclusivity, and equity-centered thinking. Table 3 includes brief overviews of several case studies, followed by a deeper review of a selected few to explore how the design methods were used and how they specifically translated into product features, user experience considerations, and outcomes.

**Table 3:** Examples of digital health tools that employ equity-centered design principles.

Digital health tool	Purpose and target population	Delivery modality	Equity-related frameworks and methods used
DIAMANTE [81]	To increase physical activity in adults with co-morbid diabetes and depression from low-income and racial and ethnic minority backgrounds	Mobile app	A user-centered design that leveraged participatory design elements was used. The target population assisted in the design and content of the mobile app design and text messages. Three phases of iterative design involved the target population in developing an app prototype, developing content, and usability testing.
eSeniorCare [82–84]	To promote successful aging in low-income older adults with multiple chronic conditions.	Mobile app	A community collaboration project that utilized action research, participatory design workshops, and user-centered design approaches to design and develop a mobile health solution that was accessible and meaningful to an aging population for self-management of their health conditions.
FAITH! App [85–87]	To improve cardiovascular health among African Americans in the Midwest by addressing the American Heart Association’s Life’s Simple 7 factors	Mobile app	Community-based research including formative feedback on initial concept, iterative prototyping, and ongoing engagement with church leaders and end users to refine content and approach.
Imi [88] by Hopelab	To support mental health and wellbeing among LGBTQ+ youth	Web-based app	Established the Hopelab Co-Creation Collective to work directly with members of the target audience to understand needs, co-create intervention content, and refine the intervention over time.
JoyPop app [89,90]	To increase mental resilience among Indigenous youth	Mobile app	Consistent with IDEAS, conducted pre- and post-development assessments with members of the target audience, including system-involved youth, and collaborated with clinician-scientists with expertise in trauma and resilience.
MiSalud [91]	To increase access to evidence-based health information and healthcare among Hispanic and African American adults.	Mobile app	Community based participatory and co-design research with adults from the target populations. For example, the app was developed with direct input from community members, and a 3-phase co-design process was used that engaged participants in multiple aspects of app development and evaluation of the cultural appropriateness.
MyStrengths [92–94]	To help people with chronic conditions identify and leverage their personal strengths in their daily lives.	Mobile app	Community-based research that included influencer workshops, expert-led seminars, co-design workshops including end users, and prototype evaluation by end users.
Pointing Interaction Notifications and Adaptations (PINATA) [95]	To understand privacy expectations and other privacy requirements for emerging technologies used with older adults.	Product workshop	Participatory design workshops used to understand end user preferences, expectations, and needs with respect to privacy and other functional aspects of emerging technologies.
Precision Nudging [45,96]	Personalized communication to drive healthcare utilization.	Text message, email, and other communication modalities	The Double Diamond method was used to develop intervention content, focusing on the needs of underserved groups to ensure inclusion of appropriate content. Personalization technology selects optimal content for individual recipients.
PREVENT Tool [97]	To improve health behavior counseling for patients of higher weight at the point of care.	Website; EHR	Participatory co-design with healthcare teams and community-based organizations in urban and rural communities.
PROCare4Life [98]	To improve quality of life for older adults with dementia or Parkinson’s disease by supporting them and their caregivers and providers.	Web and mobile app	The discover and design phases of the Double Diamond model were used to develop product requirements by soliciting unmet needs from patients, caregivers, and healthcare providers.
Skylight [99]	Uses a focus on spirituality to improve mental health among GenZennials	Web and mobile app	In the integrate phase of the IDEAS framework, gathered insights about user needs via landscape assessment, survey, and interview, and aligned with behavioral theories of mental health and stress management.

## Case studies using the Double Diamond model and IDEAS framework

Recall that the Double Diamond model alternates between divergent and convergent phases of work across a broad set of underlying research and design activities. The IDEAS framework fits the broad constraints of the Double Diamond but includes the application of behavioral theories as a necessary design step. The following case studies demonstrate how the Double Diamond model and IDEAS framework can be applied to the design of digital tools to support health equity.

### PROCare4Life

Personalized Integrated Care Promoting Quality of Life for Older People, or PROCare4Life, is a personalized integrated care platform for older people living with dementia or Parkinson’s disease. Older age is a dimension often associated with reduced health equity [26]. The first 2 stages of the Double Diamond model were used in the product design process [98,100], with multiple research methods brought to bear. This process was deliberately undertaken with an eye to equity for aging, as demonstrated by advance publication of a research protocol outlining objectives including understanding the daily lives and needs of end users [100].

First, in support of the discover phase, the designers used surveys, semi-structured interviews and workshops to gather broad insights and data on needs and contexts of end users including patients, caregivers, and healthcare professionals, who were recruited with an eye to diversity. Then, in the define phase, preliminary product requirements including content and feature suggestions and technological capabilities were developed from qualitative insights and quantitatively assessed to determine their relative importance. While patients were most interested in the ability to monitor symptoms, their caregivers ranked detection of adverse events as a top value driver.

By using multiple methods at each stage, the researchers were able to thoroughly investigate the complex landscape of user requirements for multiple audiences, setting a solid foundation for the subsequent stages of the design process. The efficacy of the digital PROCare4Life tools will be measured in a randomized control trial [101]. It is, however, notable that the PROCare4Life design team comments on the difficulty of reconciling the needs of different user groups for a project like this one and cautions that prioritizing the needs of one group, such as patients, may be necessary [98].

### Precision Nudging

Precision Nudging is a personalized intervention that uses a type of AI called behavioral reinforcement learning (BRL) to select specific messages to send to people to get them to take action on health behaviors recommended by their providers or other healthcare professionals [102]. Each Precision Nudging intervention has a set of defined target behaviors, such as scheduling and attending a mammogram screening or scheduling and completing a vaccination appointment, that the BRL algorithm is optimized to achieve by sending messages that resonate for the recipient. The available messages are created by behavioral designers to address specific determinants of the target behaviors as identified in primary and secondary research.

The Precision Nudging team used the Double Diamond model as the foundation of their approach [45]. For example, in developing an intervention to promote vaccination against COVID-19, the design team set a goal to ensure the needs of groups including black and Hispanic Americans and people with lower SES who are historically under-served by vaccination campaigns were met. In the discover phase, designers broadly assessed determinants of vaccination for people of different races, ethnicities, and SES levels through literature review; in the define phase, they prioritized determinants for inclusion in the intervention with a focus on ensuring ones relevant to underserved groups were included even if fewer people overall experienced them. In the develop phase, designers created content rooted in behavior change techniques to address the selected determinants while maintaining best practices for low literacy and low health literacy populations. Finally, in the deliver phase, the BRL technology selected behavioral content based on likely relevance to intervention recipients based on their characteristics and prior behaviors. An analysis of SMS replies from recipients of the COVID-19 vaccination intervention suggested that black recipients were especially likely to have favorable responses to the messages [103].

A similar approach was used to create a Precision Nudging intervention for mammography that was then tested in a diverse health system population of 139,164 women overdue for their mammogram. The results suggested that the approach yielded equitable outcomes, with roughly proportional rates of mammogram completion among women of different ages, races, educational attainment, or household income levels [104].

### Skylight

Skylight is a spiritual self-care app focused on mental health for people born between 1995 and 2012, collectively known as GenZennials. Released in 2020, Skylight aims to cultivate an inclusive space for people of different faiths and backgrounds and features practices such as prayer, yoga, meditation, affirmations, and music. Skylight was designed to be accessible to those from diverse racial/ethnic backgrounds, gender identities, sexual orientations, and SES, and incorporated deliberate health equity design steps as part of continuous iteration on the initial prototype. Skylight is free to use, mitigating the financial barriers users may have to accessing a self-care app to manage their mental health. Skylight’s designers have taken intentional steps to design an equity-centered intervention for efficacy at scale by using the IDEAS framework. Specifically, the designers used the integrate phase of the framework by conducting extensive research to understand user needs grounded in context and to align those needs with behavioral theories.

In order to understand the current landscape and context for GenZennials’ use of digital mental health tools incorporating spirituality, the researchers first conducted a scoping review of existing tools designed specifically for GenZennials and found a lack of such tools in the market [105]. The practitioner team created the prototype Skylight app based on their initial market research. Then, they gathered insights directly from GenZennial users of the prototype app via a cross-sectional survey [99]. At this stage, behavioral theory was woven into the interpretation of user feedback to understand associations between use of the Skylight app, mental health, sleep, and spiritual well-being. Then, more in-depth insights from users were gathered via interviews to understand perceptions of the Skylight app and the ways in which the app supported users’ perceived spirituality. These inquiries were deliberately structured to be inclusive of diverse spiritual and religious backgrounds, with a focus on improvements to Skylight that make it more broadly relevant to a variety of faiths [106].

Skylight’s equity-centered approach incorporates the voices of the intended audience via feedback in survey and interview format. Users provided insights on their experiences with the app and desired future functionality. The Skylight team is currently adding more content per recommendations from users, including more inclusive and representative content from LGBTQ+, male, and multilingual creators to better support the target audience. Additionally, Skylight is adding other content delivery modalities (e.g., YouTube) that are more popular with GenZennials. Finally, Skylight is using the data collected to inform AI that will help meet individual needs via a personalized spiritual self-care practice experience.

## Case studies using community collaboration methods

The digital tools in this section were all developed using some form of community collaboration, ranging from targeted participatory design activities to long-term partnerships with community organizations to support development and implementation.

### Imi

Imi is a digital web-based intervention using cognitive, behavioral, and identity affirmation strategies to improve mental health among LGBTQ+ youth. The self-guided, asynchronous intervention includes educational modules, exercises, and multimedia content designed to help LGBTQ+ youth explore and affirm their gender identity and sexual orientation and learn skills for coping with stress and stigma. Imi was developed by Hopelab, a nonprofit social innovation lab, in partnership with CenterLink, a coalition of over 300 LGBT centers internationally.

Prior to development, Hopelab conducted formative work including surveys, focus groups, and interviews with LGBTQ+ youth to identify intervention needs and preferences. They focused primarily on soliciting the input of people from racial and ethnic minority backgrounds, who are underrepresented in LGBTQ+ intervention research [107]. The evidence-based clinical strategies selected to address minority stress and affirm identity were informed by reviewing the scientific literature under the guidance of scientific advisors.

Then, participatory design and co-design strategies were used to develop imi [88]. Hopelab established the Hopelab Co-Creation Collective (or Youthlab), which included a racially and ethnically diverse group of LGBTQ+ youth [108]. The group held co-design workshops and sessions where Youthlab members worked alongside the Hopelab team to co-create intervention content. Hopelab also solicited feedback from key community partners, including scientific advisors, LGBTQ+ organizations, and industry associates, to refine imi. A pilot randomized control trial evaluating imi’s acceptability and effectiveness showed that participants showed greater satisfaction, improvements in coping skills, and belief in their ability to cope compared to individuals who received a control intervention [88], suggesting good initial outcomes.

Imi’s creators displayed equity-centered thinking by positioning LGBTQ+ youth feedback as the starting point for intervention development. Further, their focus on youth from racial and ethnic minority communities signifies their commitment to developing an intervention that would be acceptable and culturally relevant for a wide array of youth, and thus more likely to mitigate health disparities. Equity-centered thinking was also evident in the use of participatory co-design strategies, where LGBTQ+ youth were invited to serve alongside practitioners to create multimedia intervention content and participate in design decision-making. Finally, the intervention involved key influencers throughout the process, including scientific advisors to ensure that evidence-based strategies were being used, LGBTQ+ organizations to ensure cultural appropriateness and that the developed intervention could be disseminated and scaled, and industry partners to ensure that technological aspects such as privacy and usability were considered.

### Pointing Interaction Notifications and Adaptations (PINATA)

The PINATA project aimed to improve technology navigation for older adults and people with conditions such as Parkinson’s disease that affect motor control. The researchers organized participatory design workshops with older adults with low levels of technical experience [95] to inform the design of emerging technologies by eliciting end-user privacy perspectives. The workshop activities were based on a toolkit of tangible paper-based cards, charts, and prototypes developed to help nonexpert end users express their privacy preferences and expectations towards Adaptive Assistive Technologies (AATs). During the workshops, participants used the toolkit to organize health data types according to an AAT usage scenario. They then categorized cards describing different entities who might access their data according to how and how often they expected that access to occur. Then, participants used a Wheel of Emotions tool to articulate their feelings if their expectations about third-party data access were not met. Finally, participants selected how they wanted to enforce their expectations using Privacy Standard Strips describing standards like HIPAA and GDPR in nontechnical terms.

These workshops illustrate how to adapt technically complex contexts to empower nontechnical participants to express concerns and preferences. This approach advances equity-centered thinking by incorporating the voices of all users, not just the privileged audience with technical expertise. The activities and tools used in the workshops allowed participants to express their feelings and expectations in ways that were familiar and meaningful to them. This flexibility offered by the design activities ensured that participants could contribute regardless of their communication style or ability.

The researchers also noted opportunities for improvement. Participants had difficulties with some tools, highlighting the need to select activities that resonate with the target audience. Additionally, the workshops revealed that one-time, cross-sectional interactions might not sufficiently capture dynamic perspectives as people gain experience with the target behaviors. Long-term engagement may better support equity over time.

### FAITH! app

The FAITH! app adapts the American Heart Association’s Life’s Simple 7 framework to be culturally relevant to African Americans (AA) in the Midwestern United States [85], in recognition that African Americans experience elevated cardiovascular risk and relatively poor scores on these measures [109]. In order to ensure the AA community was centered in the design, the intervention was co-designed with community influencers under the guidance of a diverse Steering Committee [110]. The FAITH! intervention was initially intended to be used in community settings where it was found feasible and acceptable. More recently, the team behind FAITH! used a community-based participatory research process to test and refine the intervention [87].

The first phase of community-based design work was insight gathering via focus groups with AA community members and church partners to understand potential end-user preferences for FAITH!. The practitioner team synthesized those insights to outline basic app features while also incorporating behavioral theories from their conceptual framework. They collaborated with graphic designers and software developers to create the preliminary app prototype and content. This prototype was then submitted for community partner review in meetings with church leaders. The selected community partners reviewed the semi-functional prototype, testing features and usability, providing feedback on alignment with the culture of the local AA faith community, and informing subsequent iterations. The practitioner team also worked with community members on implementation considerations such as recruitment strategy, data collection methods, and long-term success metrics. From there, the team developed a functional minimum viable product (MVP) for community pilot testing; results suggested both good engagement and improvements in targeted cardiovascular behaviors [87].

The community-based participatory design process used to develop the FAITH! app demonstrates equity-centered thinking in several ways. AA community members and church partners were actively involved in each phase of the design process, ensuring that their voices and needs were central and prioritized. There was also a deliberate effort to tailor the intervention to the cultural context of the AA faith community by incorporating their cultural values and practices into the app design. Moreover, there was a focus on the usability and accessibility of the mobile app for the target community, including considerations around technology use patterns and preferences. The researchers ensured that the intervention was not only culturally relevant but also accessible to those who might benefit from it.

## Discussion

In this paper, we call on digital health practitioners to bring an explicit focus on health equity to their work via deliberate infusion of equity considerations into design methodologies. We recommend they begin by evaluating which determinants of health equity are most relevant for their target population. This can be achieved by using frameworks such as PROGRESS-Plus, eHLF, DHF, and HEIF to understand end users, and the context and way in which a digital health tool will be used. These frameworks help practitioners identify specific dimensions to consider in their digital health tool, such as race, SES, access to technology, historical inequities, or community relationships. Each framework focuses on a different aspect of equity with some shared themes such as literacy considerations and accounting for the context in which people live. Therefore, designers may need to consult multiple frameworks to ensure they address the relevant equity domains. The frameworks described in this paper are not exhaustive; others, such as ConNECT [111], may further enrich designers’ viewpoints on how to address health inequity.

Once designers have focused on a population and problem space and understood the key equity dimensions at play for their work, they must choose methods to operationalize health equity. The design world offers a rich toolkit of options that can be infused with an equity focus by designers. These include versions of the Double Diamond model, the IDEAS framework and toolkit, and a variety of community collaboration techniques including participatory design. A characteristic shared by these and other methods is the need for the design team to ensure they are using the tools in support of health equity, as the tools themselves are topic neutral. Attention to the equity dimensions identified at the outset of the design project is a must, with intentional focus on how the needs of underserved groups can be understood and met.

This paper includes several examples of digital health tools that were designed with focus on equity within diverse populations such as older adults, LGBTQ youth, African Americans churchgoers, and adults with special needs. The solutions targeted diverse health needs including emotional well-being and mental health, coping with health conditions such as dementia, Parkinson’s disease, and cardiovascular disease, or the promotion of spiritual well-being. The digital health solution case studies used a variety of tools including the Double Diamond model, IDEAS framework, and community-based design methods, with an explicit focus on incorporating an equity focus to those methods.

Community partnerships can help make equity-focused research more successful. Community organizations can act as intermediaries in logistical arrangements, potentially solving issues related to geographical isolation or lack of resources. They can assist in organizing community meetings at convenient times and locations, making it easier for individuals with work commitments to participate. These organizations can also help articulate the immediate and long-term value of participating in the design process, thereby encouraging active community involvement. For practitioners working in academic settings, most research-active universities and academic medical centers support community advisory boards or community engagement principles. Such organized research supports can help directly link investigators to leadership in appropriate community-based organizations (e.g., faith-based institutions, community health centers) serving the populations of interest [112].

The selection of which specific design methods to use is often driven by practical considerations, as illustrated in the case studies of equity-focused digital health tools. The level of collaboration of the FAITH! app team with the AA church community, or Hopelab with LGBTQ+ youth, represent gold standards in soliciting input from and involving end users as co-designers. It was vital that the creators of the FAITH! app work with community church leaders both to ensure appropriate cultural tailoring and to facilitate community access to the app and gain the trust of end users. And as a social innovation lab, it is within Hopelab’s mission and purview to deeply embed with the young LGBTQ+ community in the creation of digital health tools that address their needs. The choice of methods may also be driven by considerations like product maturity; the researchers working on PINATA leveraged paper-based tools to understand user needs for a nascent technology, while Skylight solicited feedback on a working version of the app.

These case studies also illustrate how health equity considerations can be brought to bear at different stages of product maturity. To achieve equity-centered design, it is never too late or too small. Case studies demonstrate some designers starting with simple steps to equity-centered design while building their product, and some initiating their equity-centered design steps in later stages, integrated into subsequent iterations of their product. The Hopelab team, for example, incorporated end users as co-designers from the outset, while the Skylight team began soliciting direct input from end users after developing an initial prototype. The ability to shift toward a greater equity focus over the life of a product is promising for teams who have met challenges including such considerations in their initial work.

It is also possible to make small initial forays into equitable design. For first-timers, trying a few more accessible equity-centered design steps could provide better data about the cost, value, and potential challenges of these steps to win continued support and work more efficiently. The Double Diamond methods offer one such entry point by allowing for inclusion of secondary research, which can be a benefit for resource-constrained teams or those with limited access to community users. If practitioners haven’t incorporated equity in their solution, it’s not too late.

There are many benefits to equity-focused design including the establishment of partnerships, the development of trust and empathy with target audiences, and creating a space for conversation about inequity and its causes [113]. Collectively, interventions that are developed with community participation and input at each stage are more likely to engender trust that facilitates their uptake, effectiveness, and impact. While there are challenges associated with these design approaches, the benefits are compelling and suggest the effort is worthwhile.

## Designing for implementation

While an in-depth discussion of implementation strategy was beyond the scope of this paper, it bears mentioning that consideration of implementation must be included in the design process. Digital health interventions cannot be successful without being *designed* to support implementation and future dissemination “out in the wild” [114]. The determinant frameworks mentioned above, particularly the HEIF, incorporate aspects of context to facilitate digital health designs that will fit the settings in which they will be used. As previously mentioned, the list of frameworks is not exhaustive and additional implementation science frameworks (e.g., Consolidated Framework for Implementation Research (CFIR) [115]; the Practical Robust Implementation and Sustainability Model (PRISM) [116]) can be leveraged to support the understanding of context. Beyond considering the end user and the immediate context in which the digital health tool will be used, it is important to also consider how organizational and societal contexts may influence equitable widespread adoption and sustainability of the product. For example, it is possible digital interventions targeting rural individuals may require design features that overcome the societal context of limited, unreliable internet availability in these regions. Using participatory methods to design for dissemination, scale, and sustainability (including ongoing support from community members), is necessary to ensure the tool equitably reaches and continues to be used by the target audience to reduce health disparities [117]. Designing for dissemination and sustainability should include partners beyond the end user, such as organizational leadership, payers, and policy makers, who may positively or negatively influence the widespread, long-term use of the tool.

Measurement of whether digital health tools successfully addressed disparities is critical to understand the implications of these equity-centered design processes. A systematic review found 5 main methods of assessing digital health equity, including (1, 2) descriptions of reporting and analysis in both systematic reviews and primary research; (3) assessing effects (and effect sizes) across sociodemographic factors; (4) applicability assessment of tools against under-served populations; and (5) influencer/expert assessment [118]. A scoping review of the impact of human centered design processes on health equity found most inquiries focused on immediate impact on research participants (e.g., satisfaction), rather than behavioral or health outcomes associated with any resulting tool [24]. Assessments of whether digital health tools achieve health equity are, at best, inconsistently performed at present time and represent a major area of opportunity. Furthermore, using frameworks such as RE-AIM to evaluate the implementation, reach and maintenance of the digital health tool is necessary to ensure the tool reaches your intended audience and continues to generate the intended impact [119]. These measurement strategies should be planned from the outset.

## Limitations and opportunities

There are of course challenges to successfully executing an equity-focused design process. A major limitation is access to community members. From a practical perspective, it can be expensive and time-consuming to engage community members in co-design—not just for the sponsoring organization, but also potentially for the community members themselves. It may be necessary for designers to find ways to collaborate outside of typical working hours or in locations convenient to the community to minimize disruption to participants’ routines. It can also be challenging to establish a new relationship with a community organization or leaders, or to maintain existing ones over time [120]. Yet, these relationships can be critical to the success of the digital health tool.

There are also ethical considerations to enlisting community members in design work. While a thorough discussion of the potential pitfalls is beyond the scope of this paper, several instructive reviews exist to help guide ethical practitioner behavior [121,122]. This guidance is particularly crucial when the digital tool being developed addresses vulnerable characteristics (such as having a stigmatized health condition or engaging in stigmatized behavior) where individuals could experience harm because of their participation. Other ethical concerns include how to respond emotionally to difficult disclosures or situations, ownership of research insights and resulting products [123], and how to report on research when doing so might create risk for participants [124]. Practitioner teams must thoroughly consider the potential risks of community-based methods and take steps to minimize them. An ethically conducted community design project should not only minimize risk to the community, it should confer benefits aligned with the Belmont principles [125], such as the introduction of a needed tool or upskilling community members through their participation in the project.

Another limitation to the proposed approach to improving digital health tools’ ability to deliver equitable results is that evaluating success is difficult. As noted earlier, evaluations of whether a digital health tool achieves equitable outcomes are inconsistently done and reported. Many of the tools that have been developed with a deliberate focus on health equity are also relatively new, which limits the ability to report on whether equity has been achieved on lagging indicators such as disease prevention or sustained biometric improvements (which may explain the lack of such outcomes in a recent scoping review [24]). Initial outcomes associated with these digital health tools seem positive, although time is required to accumulate evidence for the effectiveness of these approaches in achieving health equity. We hope to see an increasing number of proof points with time as these digital health tools mature and new ones are introduced.

Moreover, while the diversity of approaches accommodated within these design tools is a strength in that it makes equitable design more accessible to practitioners, it can also be a limitation in that it is difficult to establish clear standards for what appropriate usage looks like. A challenge faced during our analysis of the cases was that authors rarely mentioned the specific tools they used for designing equitably, limiting the ability to compare approaches in the current literature. Again, this is an opportunity for the field to improve as more equity-focused processes are put into practice. Given some of the ethical concerns outlined above, it is also critical to evaluate whether the process of creating these tools was equitable, which would be facilitated by direct description of those processes. We optimistically believe that the need for better and more systematic evaluations of equity-related processes and outcomes represents a major opportunity in digital health.

In particular, there is an opportunity to evaluate the different health equity frameworks and design tools described in this paper against their impact on outcomes. As more of these digital health tools are put to use, there should be an accumulation of data that permits an assessment of outcomes and a comparison of which tools might be most fit for purpose. Research may elucidate which frameworks or tools are most relevant for specific communities, target behaviors, or types of digital health tools; these findings can then guide subsequent design teams in their choice of methods.

Ultimately, we hope to see the development of a standardized evaluation checklist that facilitates design teams to understand the impact of digital health tools. Such checklists have shown promise in evaluating health equity in other domains, including adverse events in gynecologic care [126], supporting policymaker evaluation of programs [127], and evaluating global health partnerships [128]. A checklist approach could encourage and facilitate the consultation of equity frameworks early in the design process to identify equity considerations, the selection of appropriate design methods to understand user needs and tool requirements, choices about implementation methods, and the design of an evaluation strategy to assess outcomes and iteration opportunities.

## Conclusions

In the past few decades, practitioners have seen but not realized the promise of digital health tools to support diverse populations in understanding, managing, and improving their health. While technology offers a scalable way to personalize the delivery of services [129], it has been fraught with challenges ranging from the Digital Divide and lack of digital self-determination, to inadequate attention to structural or historic inequities, to commercial pressures that complicate investments in community research. However, the changing legislative and social environment in the United States has made it imperative to renew focus on ensuring that digital health tools deliver equitable experiences and outcomes to end users. We offer strategies for achieving that by considering equity dimensions via frameworks when initiating a digital health tool design project and selecting research and design methods that accommodate a nuanced consideration of end user needs. A series of case studies of existing digital health tools show how these methods have been successfully used, as well as where there are opportunities for improvement and standardization. Finally, we call on the members of our extended practitioner community to join us in highlighting health equity in their work. The time to focus on health equity in digital health is now, and we are the people to do it.
